# Clinical Outcomes and Real-World Effectiveness of Amoxicillin-Clavulanic Acid Across Indian Healthcare Settings: A Retrospective Multicenter Study

**DOI:** 10.7759/cureus.104282

**Published:** 2026-02-26

**Authors:** Anand Raju, Ajay Kakar, D K Bhagat, Sambit Kumar Behera, Rahul Pathak, Shruti Pal

**Affiliations:** 1 Ear Nose and Throat Surgery, Anand ENT Hospitals, Coimbatore, IND; 2 Periodontology and Implantology, LeVisage Clinic, Mumbai, IND; 3 Implantology, Multispecialty Advance Dental Rehabilitation and Implant Centre (MADRIC), Ranchi, IND; 4 Gastroenterology, Maharaja Krishna Chandra Gajapati Medical College & Hospital, Brahmapur, IND; 5 Medical Affairs, Indchemie Health Specialities Pvt Ltd, Mumbai, IND

**Keywords:** amoxicillin clavulanic acid, antibiotic, antimicrobial, bacterial infection, dental infection, h pylori, indications, lower respiratory tract infection, treatment, upper respiratory tract infection

## Abstract

Background

Amoxicillin-clavulanic acid is one of the most prescribed antimicrobial combinations in India. However, data on its real-world prescribing trends, effectiveness, and safety remain limited.

Objective

To evaluate prescribing patterns, dosing practices, adjuvant use, clinical effectiveness, and adverse drug reactions (ADRs) associated with Amoxicillin-clavulanic acid across Indian healthcare settings.

Methods

A retrospective, questionnaire-based multicentric study was conducted using anonymized data from 30,654 patients prescribed Amoxicillin-clavulanic acid under protocol IND/ACL/01. Parameters assessed included demographics, indications, prescriber specialty, dose, duration, adjuvant use, and outcomes. Descriptive statistics were applied.

Results

Among the analyzed prescriptions, upper respiratory tract infections (52.4%) and lower respiratory tract infections (19.5%) were the most common indications, followed by urinary tract infections (10.0%), *Helicobacter pylori (H. pylori)*-associated gastritis (6.8%), skin and soft tissue infections (5.0%), and dental infections (3.0%). Clinical resolution was achieved in 82.5% of patients, with effectiveness particularly notable in respiratory and dental infections. Adverse drug reactions, mostly mild gastrointestinal symptoms such as diarrhea and nausea, were reported in 21.6% of cases. Treatment duration averaged 7.5±2.8 days, with shorter courses showing similar clinical success. The most common regimen was amoxicillin-clavulanic acid 500/125 mg twice daily for seven to 10 days. Proton pump inhibitors (PPIs) were co-prescribed in 53.9% of cases, mainly rabeprazole, along with probiotics (23.3%).

Conclusions

Amoxicillin-clavulanic acid demonstrated high clinical effectiveness across common indications such as respiratory tract infections, urinary tract infections, skin and soft tissue infections, as well as in dental infections and *H. pylori*-associated gastritis. Clinical resolution was achieved in over 80% of cases, with a favorable safety profile characterized mainly by mild gastrointestinal adverse events in about 20% of patients. These real-world results underscore its valuable role in diverse Indian healthcare settings, supporting evidence-based prescribing for optimized outcomes.

## Introduction

The combination of amoxicillin and clavulanic acid is a cornerstone of empirical therapy for both community and hospital-acquired infections [1,2]. Amoxicillin, a semisynthetic β-lactam antibiotic, possesses broad-spectrum activity against Gram-positive and select Gram-negative organisms. However, its efficacy is often compromised by bacterial production of β-lactamase enzymes, which hydrolyze the β-lactam ring, rendering the antibiotic inactive. Clavulanic acid, a potent β-lactamase inhibitor, was developed to overcome this limitation by binding irreversibly to the active site of β-lactamase enzymes, thereby protecting amoxicillin from enzymatic degradation and restoring its antimicrobial spectrum [3].

The combination of amoxicillin-clavulanic acid has therefore been widely adopted as a first-line or empirical treatment in a variety of infections, including respiratory tract infections, urinary tract infections, skin and soft tissue infections, and odontogenic infections. Its clinical utility stems from broad coverage, an established safety profile, and availability in both oral and parenteral formulations. The irrational and indiscriminate use of amoxicillin and clavulanic acid has become a global concern. Excessive duration, suboptimal dosing, and inappropriate indication selection have all contributed to the growing challenge of antimicrobial resistance to amoxicillin and clavulanic acid [4,5]. Emerging resistance among Enterobacteriaceae, Staphylococcus aureus, and Haemophilus influenzae isolates underscores the urgent need for stewardship-driven rationalization of amoxicillin and clavulanic acid use.

The World Health Organization (WHO) classifies amoxicillin-clavulanate within the “access” category of the access, watch, reserve (AWaRe) framework, reflecting its importance as a first-line agent for the treatment of common community-acquired infections. This classification underscores its established efficacy, safety, and comparatively lower resistance potential, supporting its inclusion in standard empirical therapy protocols. WHO further emphasizes ensuring the availability and rational use of “access” antibiotics like amoxicillin-clavulanate to maintain their effectiveness and curb the rise of antimicrobial resistance globally [6].

In the Indian context, the situation is particularly complex. The combination of easy over-the-counter access, empirical prescribing habits, and variable diagnostic support has led to amoxicillin and clavulanic acid being one of the most frequently dispensed antibiotics in outpatient and inpatient care [7]. Recent Indian observational studies and clinician surveys demonstrate high and sustained use of co-amoxiclav across both primary and secondary care settings, reflecting its entrenched role in empirical prescribing practices [8]. While its broad activity offers convenience, it also raises the risk of unnecessary exposure and resistance selection. Amoxicillin-clavulanic acid is also among the antibiotics most frequently associated with antibiotic-associated diarrhea, reported in approximately 10-25% of exposed patients, likely due to disruption of the intestinal microbiota and overgrowth of enteropathogenic organisms. Consequently, probiotic co-prescription has been increasingly adopted in routine practice to mitigate gastrointestinal adverse effects and restore microbial balance [9]. Understanding real-world usage patterns, including dose, frequency, duration, indication, and associated outcomes, is therefore essential to inform evidence-based stewardship policies.

Accordingly, this retrospective multicentric analysis (Protocol IND/ACL/01) was undertaken to characterize amoxicillin and clavulanic acid prescribing practices across various healthcare settings in India. The study aimed to (1) describe prescribing trends by specialty and indication, (2) assess clinical outcomes and safety profiles, and (3) identify optimization opportunities to enhance rational amoxicillin-clavulanic acid use in both community and hospital environments.

## Materials and methods

Study design and ethical compliance

A retrospective, cross-sectional, questionnaire-based, multicentric study was conducted in accordance with the principles of the International Council for Harmonisation, Good Clinical Practice (ICH-GCP), Indian GCP (2001), and the ethical standards outlined in the Declaration of Helsinki (2013 revision). The study protocol and associated documents were reviewed and approved by an Independent Institutional Ethics Committee at Lifepoint Multispeciality Hospital, Pune (Protocol no: IND/ACL/01; approval date: April 25, 2024). The study was designed to evaluate real-world prescribing patterns, treatment outcomes, and safety profiles of amoxicillin-clavulanic acid across a diverse network of healthcare facilities in India, encompassing both urban and semi-urban regions.

Participating sites included private clinics, secondary and tertiary care hospitals, and independent physician practices. The retrospective design ensured that no intervention or modification to patient management occurred; instead, data were extracted from existing medical records and case notes documenting routine clinical care.

Objectives

The primary and secondary objectives of the study were clearly defined before data extraction to ensure uniformity and analytical focus:

The study evaluated the clinical effectiveness of amoxicillin-clavulanic acid by assessing symptom resolution, patient-reported improvement, and the frequency and nature of adverse drug reactions (ADRs). It also described amoxicillin-clavulanic acid prescribing patterns, including dose, frequency, and duration across different indications and care settings, identified common indications and co-prescribed adjuvants, and explored variations in prescribing practices by physician specialty and practice setting (clinic versus hospital). These objectives aim to provide real-world insights to support antimicrobial stewardship efforts.

Study population

The final dataset comprised 30,654 anonymized patient records in which amoxicillin-clavulanic acid had been prescribed for active infections between January and December 2023. The study included prescriptions from general practitioners, consulting physicians, pediatricians, dentists, and specialty practitioners such as ear, nose, and throat (ENT) and dermatology consultants.

The inclusion criteria encompassed patients of all ages and genders who were prescribed amoxicillin-clavulanic acid, either oral or parenteral formulation, for an active infectious condition. Only records with clear documentation of the indication, dose, duration, and clinical outcome were considered. Exclusion criteria included prescriptions written solely for prophylactic or preventive purposes, such as dental extraction prophylaxis, as well as records that were incomplete, illegible, or missing clinical outcome information. Additionally, patients with a known history of hypersensitivity to β-lactam antibiotics, including amoxicillin-clavulanic acid, were excluded from the study.

This broad inclusion approach was intended to ensure representativeness of the prescribing landscape across both community and institutional healthcare settings.

Data collection procedure

Data were collected retrospectively from digital case record forms (CRFs) using a standardized electronic data capture template developed for this study (Appendix 1). Each participating investigator was trained on data abstraction methods to ensure consistency in the interpretation of medical terms and recording of quantitative variables.

The dataset included variables covering patient demographics such as age, sex, and location of care. Prescribing details captured the indication for amoxicillin-clavulanic acid use, dose strength, frequency, route of administration, and treatment duration. Information on adjuvant therapy noted the concomitant use of proton pump inhibitors (PPIs), probiotics, antihistamines, analgesics, or other agents. Clinical outcomes were assessed based on physician or patient-reported symptom resolution, categorized as complete, partial, or none. Safety assessment involved documenting the presence and types of any adverse events temporally associated with amoxicillin-clavulanic acid use. All collected data were de-identified before analysis to maintain patient confidentiality. Quality control checks were performed to identify and rectify missing or inconsistent entries before the dataset lock.

The protocol (IND/ACL/01) was submitted to and approved by an Independent Ethics Committee (IEC) prior to initiation. The study did not involve any experimental procedure, patient recontact, or disclosure of identifiable information. Data confidentiality was maintained at all times, and no financial incentives were offered to participating investigators or institutions.

Statistical analysis

All statistical analyses were descriptive, reflecting the observational and exploratory nature of the study. Data were entered and analyzed using SPSS software, version 27.0 (IBM Corporation, Armonk, New York, USA). Categorical variables, such as indication, prescriber specialty, adjuvant use, and adverse drug reaction (ADR) types, were summarized using absolute frequencies and percentages. Continuous variables, including age and treatment duration, were reported as mean±standard deviation (SD) or median with interquartile range (IQR), as appropriate. Cross-tabulations and exploratory analyses, such as examining the relationship between dose and duration versus clinical outcomes and duration versus ADR frequency, were conducted to identify emerging trends. No inferential statistical testing was performed, as the study focused on descriptive characterization rather than hypothesis testing.

## Results

Demographics and setting

A total of 30,654 patients were analyzed; females constituted a slightly higher proportion (16482, 53.8%) compared with males (14,172; 46.2%), with a mean age of 37.6±12.1 years. The majority of prescriptions originated from clinic-based settings (23,259; 75.9%), indicating predominant outpatient use of amoxicillin-clavulanic acid. Hospital-based prescriptions accounted for 6,049 (19.7%), while 1,346 (4.4%) were from other healthcare settings (Table 1).

**Table 1:** Demographics and Healthcare Setting

Category	n	%
Female	16,482	53.8
Male	14,172	46.2
Mean Age (Years)	37.6	±12.1
Clinic	23,259	75.9
Hospital	6,049	19.7
Other	1,346	4.4

Prescriber specialty

**Figure 1:**
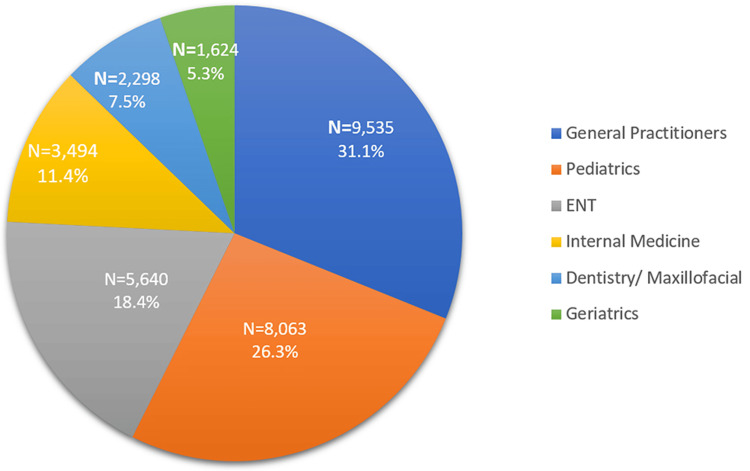
Prescriber Specialty ENT: ear, nose and throat.

General practitioners were the largest prescribers (9,535; 31.1%), followed by pediatrics (8,063; 26.3%) and ENT (5,640; 18.4%) (Figure 1).

Indications

**Figure 2:**
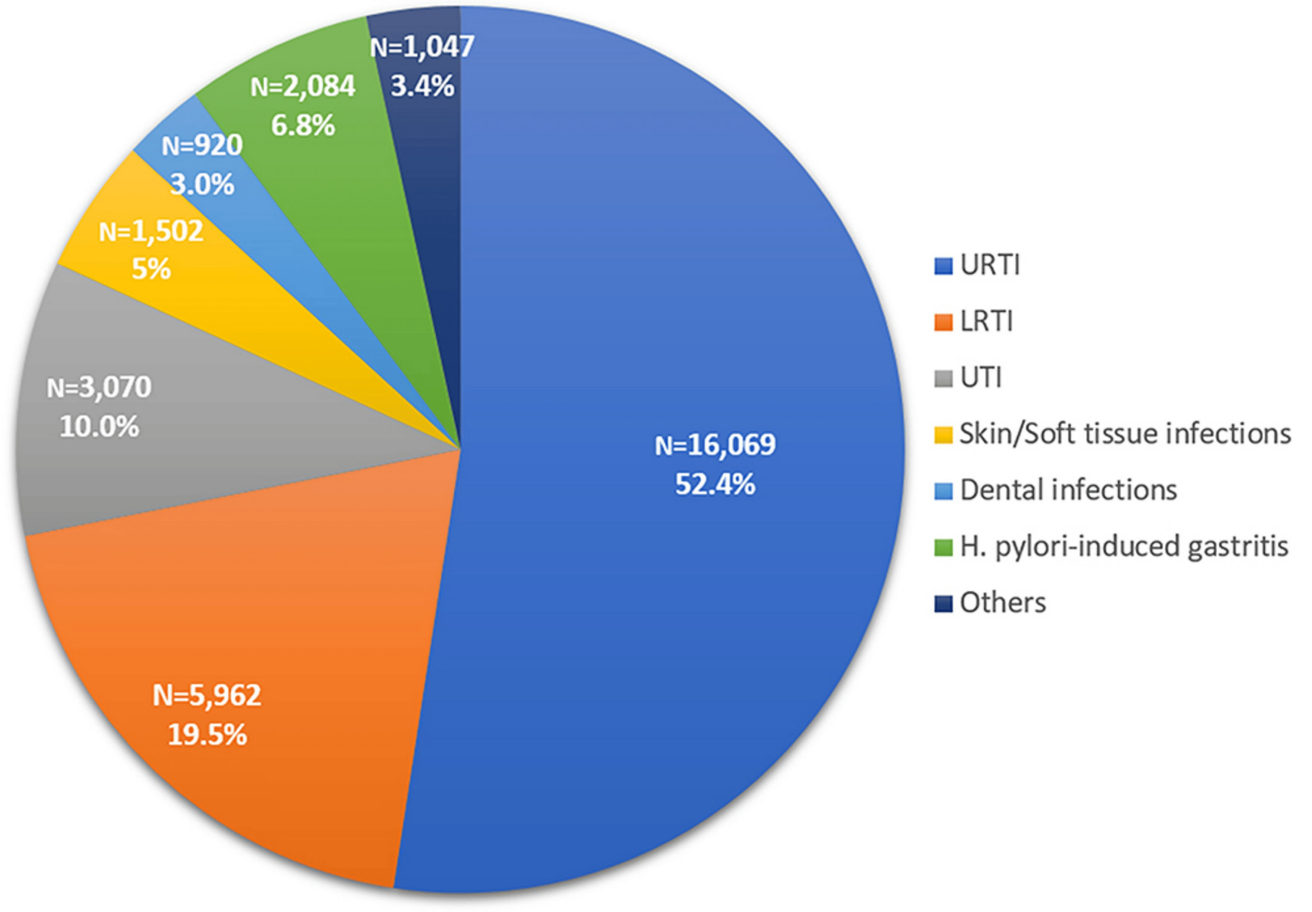
Indications for Amoxicillin-Clavulanic Acid Prescriptions URTI: upper respiratory tract infections; UTI: urinary tract infections; LRTI: lower respiratory tract infections.

The distribution of indications for amoxicillin-clavulanic acid prescriptions is summarized in this table. Upper respiratory tract infections were the most common indication, accounting for over half of all prescriptions (16,069; 52.4%), followed by lower respiratory tract infections (5,962; 19.5%). Urinary tract infections comprised (3,070; 10.0%) of cases, while skin and soft tissue infections and dental infections accounted for smaller proportions (1,502; 5% and 920, 3.0%, respectively). Notably, *Helicobacter pylori* (*H. pylori*)-induced gastritis represented 2,084 (6.8%) of prescriptions, reflecting its selective use in gastrointestinal practice (Figure 2).

Dose, duration, and frequency

The table summarizes the most frequently observed prescribing parameters for amoxicillin-clavulanic acid. The predominant dose prescribed was 500/125 mg, accounting for 91.1% of all prescriptions. A treatment duration of seven to 10 days was most commonly observed (17,951; 58.6%), reflecting standard clinical practice. Twice-daily (BID) administration was the preferred dosing frequency in 24,993 (81.6%) of cases, indicating consistency in prescribing patterns across settings. Refer to Table 2.

**Table 2:** Prescribing Patterns BID: twice-daily.

Parameter	Most common	n	%
Dose	500/125 mg	27,913	91.1
Duration	7-10 days	17,951	58.6
Frequency	BID	24,993	81.6

Adjuvant therapies

The table outlines the pattern of adjuvant therapies co-prescribed with amoxicillin-clavulanic acid. Proton pump inhibitors (PPIs) were the most frequently used adjuvants, prescribed in 16,505 (53.9%) of patients, with rabeprazole being the predominant agent, followed by pantoprazole and omeprazole. Probiotics were co-administered in nearly one-quarter of cases (7,127; 23.3%), likely to mitigate gastrointestinal adverse effects. Other supportive medications, including antihistamines, analgesics, and antidiarrheals, were prescribed less frequently, reflecting targeted adjunctive use. Refer to Table 3.

**Table 3:** Adjuvant Therapies PPI: proton pump inhibitors.

Therapy	n	%
PPIs (Total)	16,505	53.9
- Rabeprazole	9,845	32.1
- Pantoprazole	4,210	13.7
- Omeprazole	1,850	6.0
- Other PPIs	600	2.0
Probiotics	7,127	23.3
Antihistamines	2,436	8.0
Analgesics	1,624	5.3
Antidiarrheals	988	3.2
None or not available	1,974	6.4

Effectiveness and safety

The clinical outcomes observed following treatment with amoxicillin-clavulanic acid. Complete resolution of symptoms was achieved in the majority of patients (25,258; 82.5%), indicating high overall effectiveness. Partial clinical improvement was reported in 3,915 (12.8%) cases, while lack of response was uncommon (211; 0.7%). Outcome status was uncertain in a small proportion of patients (277; 0.9%), reflecting limited follow-up documentation in routine practice (Table 4).

**Table 4:** Treatment Outcomes

Outcome	n	%
Complete resolution	25,258	82.5
Partial improvement	3,915	12.8
No improvement	211	0.7
Not sure	277	0.9
Data not available	993	3.2

Specialty-wise utilization and clinical outcomes of amoxicillin-clavulanic acid. General practitioners accounted for the highest proportion of prescriptions (9535; 31.1%), primarily for respiratory, urinary, and gastrointestinal infections, with a resolution rate of 85.3%. Pediatric and ENT specialties also contributed substantially, demonstrating high and comparable clinical resolution rates of 86.2% (n=8,063) and 83.9% (n=5,640), respectively. Notably, dentistry and maxillofacial practice showed the highest resolution rate, 88.0% (n=2,298), while geriatric practice maintained consistent effectiveness of 82.5% (n=1,624), across mixed infection profiles (Table 5).

**Table 5:** Specialty-wise Usage and Clinical Outcomes of Amoxicillin-Clavulanate *Indication-wise analyses included all prescriptions with documented indication (n=30,654), whereas outcome-based analyses were restricted to records with documented clinical outcomes (n=29,661). URTI: upper respiratory tract infections; UTI: urinary tract infections; LRTI: lower respiratory tract infections.

Specialty	Primary Indications	% of Total Prescriptions	n	Resolution Rate (%)
General Practitioners	URTI, UTI	31.1	9,535	85.3
Pediatrics	URTI, Otitis media, Sinusitis	26.3	8,063	86.2
ENT	Sinusitis, Pharyngitis, Otitis media	18.4	5,640	83.9
Internal Medicine	URTI, LRTI, UTI, Gastritis	11.4	3494	84.1
Dentistry/ Maxillofacial	Odontogenic infections	7.5	2,298	88.0
Geriatrics	Mixed infections, Gastritis	5.3	1,624	82.5

**Figure 3:**
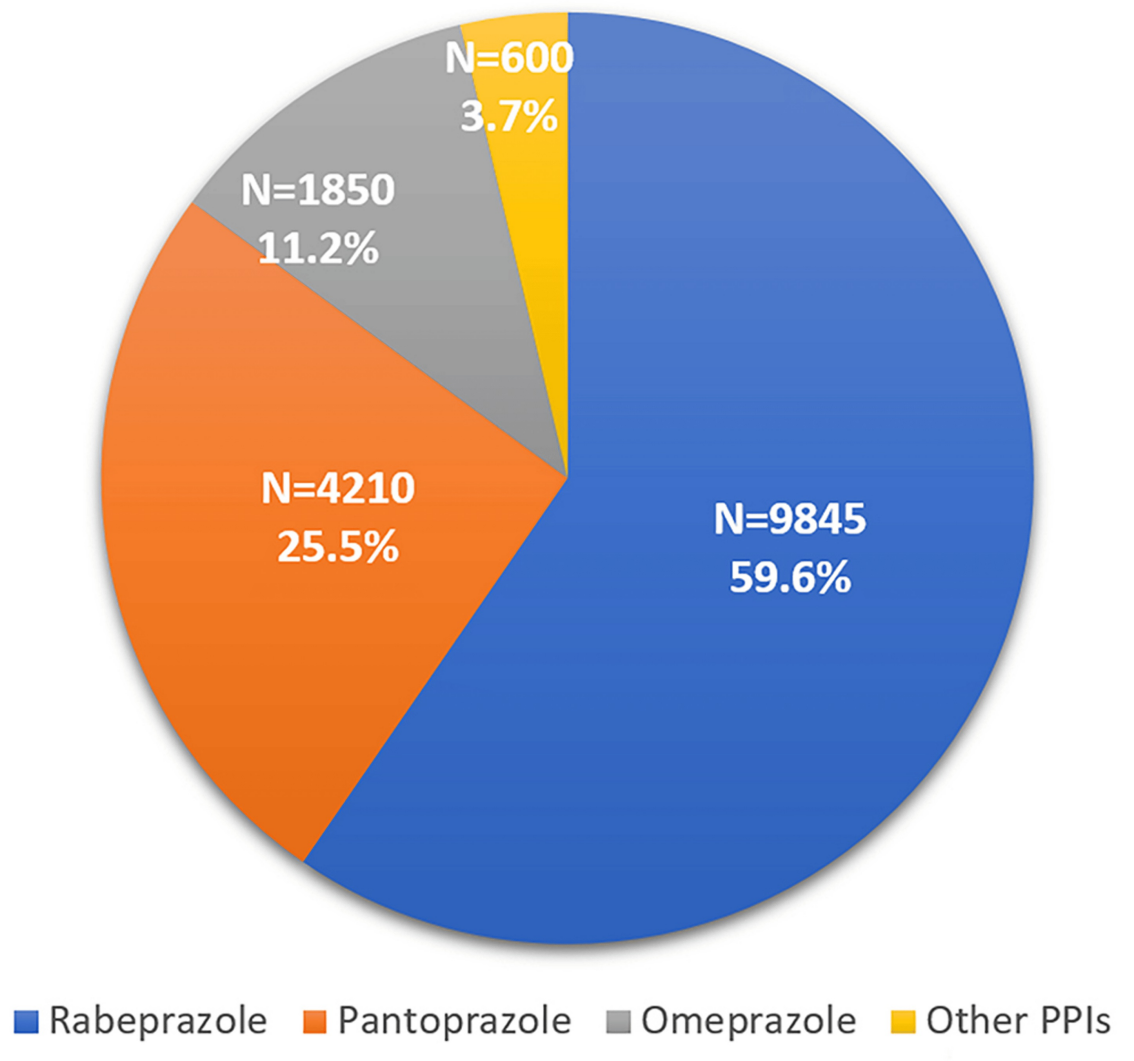
Comparative Clinical Outcomes with Different PPI Co-prescriptions PPI: proton pump inhibitors.

As shown in Figure 3, it summarizes clinical outcomes associated with different PPIs co-prescribed with amoxicillin-clavulanic acid. Rabeprazole was the most frequently used PPI (9,845; 59.6%), and rabeprazole co-prescriptions were associated with higher reported clinical resolution rates (88.4%). Pantoprazole and omeprazole showed moderate resolution rates of 82.1% and 78.9%, respectively. Other PPIs were least utilized and demonstrated comparatively lower clinical effectiveness.

The adverse drug reactions reported with amoxicillin-clavulanic acid therapy are summarized below. Gastrointestinal events were the most common, with diarrhea reported in over half of the cases (15,613; 51.0%), followed by nausea (6,676; 21.8%) and vomiting (3,263; 10.6%). Cutaneous reactions were less frequent, with rash and itching reported in 1,297 (4.2%) and 953 (3.1%) patients, respectively. Overall, the observed adverse events were predominantly mild and self-limiting in nature (Table 6).

**Table 6:** Adverse Drug Reactions

Reaction	N	%
Diarrhea	15,613	51.0
Nausea	6,676	21.8
Vomiting	3,263	10.6
Rash	1,297	4.2
Itching	953	3.1

## Discussion

This large retrospective dataset underscores the extensive real-world use of amoxicillin-clavulanic acid across India, particularly for respiratory infections, where it continues to serve as an empirically preferred β-lactam/β-lactamase inhibitor combination. The study highlights consistent clinical effectiveness and an acceptable safety profile, reaffirming amoxicillin-clavulanic acid’s therapeutic relevance in outpatient and hospital practice. The widespread use of amoxicillin-clavulanic acid observed in this analysis aligns with its broad antimicrobial coverage and historical reliability in treating community-acquired infections. Nevertheless, the findings simultaneously emphasize the need for cautious, evidence-based prescribing to preserve its efficacy amid evolving antimicrobial resistance trends.

The predominant use of the 500/125 mg twice-daily regimen for seven to 10 days in this dataset mirrors global practice patterns reported in stewardship surveillance and observational studies [10,11]. While this regimen remains clinically effective, the analysis revealed that extended treatment durations were associated with an increased frequency of adverse drug reactions (ADRs), particularly gastrointestinal events. This association highlights the necessity of optimizing therapy duration without compromising clinical outcomes.

In our study, amoxicillin-clavulanic acid accounted for 18% of all prescriptions made for ENT indications, which aligns with global and regional patterns showing its dominant role in ambulatory ENT care. For instance, the recent clinical audit from Ghana reported that amoxicillin-clavulanic acid, either alone or in combination with topical agents, was the single most prescribed antibiotic for ENT infections, contributing to nearly half of all systemic antibiotic use in their cohort [12]. The consistency between our findings and this large real-world dataset reinforces the widespread perception of amoxicillin-clavulanic acid as the empirical agent of choice for common ENT infections. However, both studies also highlight an important stewardship implication: high reliance on amoxicillin-clavulanic acid must be balanced with careful assessment of indication, guideline alignment, and opportunities to avoid unnecessary escalation.

Dental indications constituted 7.5% of all amoxicillin-clavulanic acid prescriptions in our study, aligning with evidence that co-amoxiclav serves as a targeted agent in dentistry rather than a routine first-line choice. Contemporary literature consistently identifies it as the second most used dental antibiotic, employed when broader coverage is required for β-lactamase-producing pathogens and mixed aerobic-anaerobic infections commonly seen in odontogenic abscesses and cellulitis [13]. Co-amoxiclav has demonstrated reliable activity against the full spectrum of organisms isolated from complicated odontogenic infections, supporting its role in cases such as spreading cellulitis, pericoronitis, and deep space involvement [14]. Its selective use is further supported in dental extraction settings where underlying infection or higher postoperative risk necessitates broader empirical coverage [15].

Shorter, indication-specific antibiotic courses have gained international attention as a core component of antimicrobial stewardship. Evidence from controlled trials and real-world data consistently demonstrates that abbreviated β-lactam courses (typically 3-5 days) can achieve comparable cure rates to longer regimens while reducing microbiome disruption and minimizing adverse effects [16-18]. Such data reinforce the stewardship principle that “shorter is often better,” especially for uncomplicated respiratory or urinary infections.

Our study’s findings echo this global evidence base. High response rates with shorter AMC courses, coupled with fewer reported ADRs, suggest that adopting tailored treatment durations could enhance patient safety and adherence while curbing selective pressure for resistant bacterial strains. These insights should encourage prescribers to periodically review standard amoxicillin-clavulanic acid protocols and align them with current evidence and local resistance data.

Additionally, the pattern of adjuvant co-prescription, particularly proton pump inhibitors (PPIs) and probiotics reflects clinical efforts to mitigate gastrointestinal side effects but also raises the possibility of polypharmacy. Rationalization of concurrent therapy and avoidance of unnecessary protective agents can further improve stewardship efficiency and patient compliance.

Clinical Relevance: *H. pylori* infection and amoxicillin-clavulanic acid

The role of amoxicillin-clavulanic acid in *H. pylori* eradication therapy remains a subject of ongoing debate. Traditionally, *H. pylori* treatment relies on triple therapy comprising a proton pump inhibitor (PPI), amoxicillin, and clarithromycin. However, the emergence of macrolide-resistant *H. pylori* strains has prompted the exploration of alternative or salvage regimens [19]. In this context, the addition of clavulanic acid has been hypothesized to augment eradication efficacy by inhibiting β-lactamase-producing *H. pylori* strains and enhancing the stability of amoxicillin in the gastric microenvironment [20,21].

In our real-world cohort, *H. pylori*-related gastritis accounted for 6.8% of all amoxicillin-clavulanic acid prescriptions, reflecting its selective yet clinically meaningful use in gastrointestinal practice. In this retrospective analysis, the diagnosis of *H. pylori* infection was based on the physician's clinical judgment and documented medical records. Accordingly, clinical resolution in this subgroup reflects symptomatic improvement as recorded in routine practice, rather than confirmed eradication. These prescriptions were frequently co-administered with PPIs (53.9%), predominantly rabeprazole, followed by pantoprazole, omeprazole, and esomeprazole. Rabeprazole-based combinations were associated with the highest rates of reported symptomatic resolution (approximately 85%), which may relate to its potent and sustained acid suppression, lower dependence on CYP2C19 metabolism, and potential enhancement of antibiotic bioavailability. Beyond gastric manifestations, the presence of *H. pylori* has also been explored in relation to various oral lesions, highlighting its broader clinical relevance across gastrointestinal and oral health contexts.

In addition to these real-world observations, emerging clinical and mechanistic evidence suggests that the incorporation of clavulanic acid may enhance the efficacy of amoxicillin-based *H. pylori* eradication regimens, particularly in the setting of rising antimicrobial resistance. A randomized clinical trial by Ojetti et al. demonstrated significantly higher eradication rates with an amoxicillin-clavulanate-containing triple regimen compared with amoxicillin alone, without an increase in adverse effects. This benefit is biologically plausible, as β-lactamase inhibitors have demonstrated intrinsic activity against *H. pylori* and may help overcome reduced susceptibility to amoxicillin. [20]

Additional support for this approach comes from contemporary expert guidance. The American Gastroenterological Association has acknowledged that the addition of clavulanic acid to amoxicillin-based regimens may improve eradication success by approximately 10-20%, particularly in refractory or previously treated infections, although larger confirmatory studies are warranted [22]. Similarly, evidence from Asian clinical studies and in vitro analyses indicates that β-lactamase inhibitors such as clavulanate can modestly enhance eradication efficacy in clarithromycin-resistant populations. In a comparative study from Eastern Taiwan, the LAcR regimen, comprising levofloxacin, amoxicillin/clavulanate, and rabeprazole, achieved significantly higher eradication and symptom resolution rates than standard clarithromycin-based triple therapy (78.1% vs. 57.5%, p<0.05), underscoring the therapeutic relevance of co-amoxiclav-containing regimens in resistance-tailored treatment strategies [23]. While current American College of Gastroenterology guidelines continue to recommend reserving amoxicillin-clavulanic acid for rescue or resistance-guided therapy, the convergence of real-world data, randomized clinical evidence, and mechanistic rationale supports its consideration as a rational alternative or adjunctive option in selected *H. pylori* cases [24].

To optimize outcomes and support antimicrobial stewardship, incorporation of microbiological confirmation, resistance-aware regimen selection, and adherence to updated treatment protocols remain essential to ensure rational and targeted antibiotic utilization.

Strengths and limitations

This study has several notable strengths. The inclusion of a large, multicentric sample encompassing over 30,000 prescriptions provides a robust overview of current amoxicillin-clavulanic acid prescribing behaviors in real-world Indian healthcare. The diversity of participating prescribers, from general practitioners to tertiary specialists, ensures a representative picture of national prescribing dynamics. Additionally, the study offers valuable insights into practical outcomes, adverse event patterns, and adjuvant use, all of which can inform policy and clinical decision-making.

However, some limitations should be acknowledged. The retrospective and observational design limits causal inference, and the absence of microbiological culture or sensitivity data precludes confirmation of pathogen-specific efficacy. Moreover, the reliance on physician-reported or record-based ADRs introduces the possibility of reporting bias and underestimation of mild events. Despite these limitations, the consistency of findings with global literature strengthens the validity and generalizability of the observations.

## Conclusions

Amoxicillin-clavulanic acid continues to serve as a cornerstone of empirical therapy in India, demonstrating broad clinical effectiveness and an acceptable safety profile across respiratory, urinary, and soft-tissue infections. Its shorter treatment courses are well tolerated without compromising outcomes. The observed use in *H. pylori*-associated gastritis further highlights its therapeutic versatility and potential value in resistant infections when guided by microbiological evidence. Overall, these findings emphasize the importance of rational, evidence-based use of amoxicillin-clavulanic acid in alignment with antimicrobial stewardship principles and evolving resistance patterns to sustain its long-term clinical relevance.

## Appendices

Appendix 1

**Table 7:** Case Report Form

Case Report Form	
Study Title	Exploring Amoxicillin and Clavulanic Acid Prescribing Practices: A Retrospective Analysis
Protocol Number	IND/ACL/01
Sponsor	Indchemie Health Specialities Pvt. Ltd.
Section 1: Patient Identification	
Parameter	Details
Patient Number	
Patient Initials	
Date of Enrollment	
Age (years)	
Gender	☐ Male ☐ Female ☐ Other
Relevant Medical History	
Section 2: Indication for Amoxicillin + Clavulanic Acid Prescription (Tick all that apply)	
Indication Category	Options
Upper Respiratory Tract Infections (URTI)	☐ Pharyngitis ☐ Sinusitis ☐ Otitis Media
Lower Respiratory Tract Infections (LRTI)	☐ Yes ☐ No
Urinary Tract Infections (UTI)	☐ Yes ☐ No
Skin & Soft Tissue Infections (SSTI)	☐ Yes ☐ No
Dental / Odontogenic Infections	☐ Yes ☐ No
Intra-abdominal Infections	☐ Yes ☐ No
Helicobacter pylori–associated gastritis	☐ Yes ☐ No
Mixed Infections (specify)	
Other Bacterial Infections (specify)	
Section 3: Prescription Details	
3.1 Dosage Prescribed	
Strength	Tick
250/125 mg	☐
500/125 mg	☐
875/125 mg	☐
1000/125 mg	☐
3.2 Duration of Therapy	
Duration	Tick
3–5 days	☐
7–10 days	☐
14 days	☐
21 days	☐
3.3 Frequency of Administration	
Frequency	Tick
Once daily	☐
Twice daily	☐
Three times daily	☐
Four times daily	☐
Section 4: Rationale for Choosing Amoxicillin + Clavulanic Acid	
Reason	Tick
Broader spectrum of activity	☐
Lower cost	☐
Fewer side effects	☐
Easier dosing regimen	☐
Section 5: Concomitant Medications	
Parameter	Details
Any other medications prescribed concurrently?	☐ Yes ☐ No
If Yes, specify	
Section 6: Follow-Up and Treatment Response	
Parameter	Details
Date of Follow-up Visit	
Treatment Response	☐ Completely resolved ☐ Partial improvement ☐ No improvement ☐ Not sure
Section 7: Safety and Adverse Events	
7.1 Any Adverse Effects Noted?	
Parameter	Details
Any adverse effects reported?	☐ Yes ☐ No
If Yes, specify	
7.2 Adverse Drug Reactions (Tick all that apply)	
Adverse Reaction	Tick
Nausea	☐
Vomiting	☐
Diarrhea	☐
Rash	☐
Itching	☐
Section 8: Prescribing Trends – Adjuvant Therapies	
Adjuvant Therapy	Tick / Specify
Proton Pump Inhibitors (PPI)	☐ Rabeprazole ☐ Pantoprazole ☐ Omeprazole ☐ Other: ____
Antihistamines	☐
Analgesics	☐
Probiotics	☐
Antidiarrheal agents	☐
Other antibiotics	☐ Macrolides ☐ Cephalosporins ☐ Tetracyclines ☐ Fluoroquinolones ☐ Aminoglycosides
Section 9: Healthcare Setting	
Parameter	Details
Type of Healthcare Facility	☐ Hospital ☐ Clinic
Specialty of Prescribing Physician	
Section 10: Additional Comments	
Remarks	
